# Quantitative Trait Locus Mapping for Resistance Against *Pyrenopeziza brassicae* Derived From a *Brassica napus* Secondary Gene Pool

**DOI:** 10.3389/fpls.2022.786189

**Published:** 2022-02-04

**Authors:** Chinthani S. Karandeni Dewage, Katherine Cools, Henrik U. Stotz, Aiming Qi, Yong-Ju Huang, Rachel Wells, Bruce D. L. Fitt

**Affiliations:** ^1^Centre for Agriculture, Food, and Environmental Management Research, School of Life and Medical Sciences, University of Hertfordshire, Hatfield, United Kingdom; ^2^Rothamsted Research, Harpenden, United Kingdom; ^3^John Innes Centre, Norwich Research Park, Norwich, United Kingdom

**Keywords:** crop losses, fungal pathogens, host resistance, light leaf spot, oilseed rape, quantitative resistance

## Abstract

Use of host resistance is the most economical and environmentally safe way to control light leaf spot disease of oilseed rape (*Brassica napus*). The causal organism of light leaf spot, *Pyrenopeziza brassicae*, is one of the most economically damaging pathogens of oilseed rape in the United Kingdom and it is considered to have a high potential to evolve due to its mixed reproduction system and airborne ascospores. This necessitates diverse sources of host resistance, which are inadequate at present to minimize yield losses caused by this disease. To address this, we screened a doubled haploid (DH) population of oilseed rape, derived from a secondary gene pool (ancestral genomes) of *B. napus* for the introgression of resistance against *P. brassicae*. DH lines were phenotyped using controlled-environment and glasshouse experiments with *P. brassicae* populations obtained from three different geographic locations in the United Kingdom. Selected DH lines with different levels of resistance were further studied in a controlled-environment experiment using both visual (scanning electron microscope – SEM) and molecular (quantitative PCR) assessment methods to understand the mode/s of host resistance. There was a clear phenotypic variation for resistance against *P. brassicae* in this DH population. Quantitative trait locus (QTL) analysis identified four QTLs with moderate to large effects, which were located on linkage groups C1, C6, and C9. Of these, the QTL on the linkage group C1 appeared to have a major effect on limiting *P. brassicae* asexual sporulation. Study of the sub-cuticular growth phase of *P. brassicae* using qPCR and SEM showed that the pathogen was able to infect and colonise both resistant and susceptible Q DH lines and control *B. napus* cultivars. However, the rate of increase of pathogen biomass was significantly smaller in resistant lines, suggesting that the resistance segregating in this DH population limits colonisation/sporulation by the pathogen rather than eliminating the pathogen. Resistance QTLs identified in this study provide a useful resource for breeding cultivar resistance for effective control of light leaf spot and form a starting point for functional identification of the genes controlling resistance against *P. brassicae* that can contribute to our knowledge on mechanisms of partial resistance of crops against pathogens.

## Introduction

Host plant resistance against pathogens is an important characteristic in agricultural crops. In general, resistance against pathogens is described under two broad categories: complete/qualitative resistance and incomplete/quantitative resistance (Roux et al., 2014; French et al., 2016). Of these two categories, quantitative disease resistance (QDR) is preferred as a broad-spectrum, durable source of resistance (Poland et al., 2009; French et al., 2016). Development of genetic linkage maps with DNA-based markers and quantitative trait loci (QTLs) mapping by exploiting the marker-trait associations have proved to be effective in breeding for plant disease resistance (Poland et al., 2009; St. Clair, 2010). Further dissection of resistance loci and characterisation of underlying genes can improve understanding of the mechanisms of QDR against plant pathogens (French et al., 2016), especially for those with complex host-pathogen interactions (e.g., extracellular pathogens) where the operation of host resistance does not eliminate the pathogen.

Light leaf spot (LLS), caused by ascomycete extracellular fungal pathogen *Pyrenopeziza brassicae* Sutton and Rawlinson (anamorph *Cylindrosporium concentricum* Grev.) (Rawlinson et al., 1978) is one of the most widespread diseases of oilseed rape (*Brassica napus* L.) in the United Kingdom, causing major yield losses (CropMonitor, 2016). The pathogen affects most aerial parts of the plant, including leaves, stems, flowers, and seed pods, resulting in reduction of leaf photosynthetic area, reduced plant vigour, and further yield loss through pod shatter (Boys et al., 2007). According to recent reports, *P. brassicae* can cause up to 30% yield reduction (AHDB, 2021). However, earlier studies have reported yield losses as great as 50% under severe epidemics (Rawlinson et al., 1978). Current recommendations for managing LLS risks include application of fungicides, growing cultivars with good field resistance, and crop sanitary practices, such as ploughing crop debris, delaying of the sowing date, and separation of oilseed rape crops in space and time (AHDB, 2021). In practice, the effectiveness of fungicide control methods depends on several factors, including fungicide application timings, weather, and shifts in *P. brassicae* populations toward fungicide insensitivity. Additionally, fungicide applications may not always be an economically viable solution for farmers. Despite crop sanitary practices, cruciferous vegetables, weeds species, and volunteer oilseed rape plants that occur from pod shatter during harvest can provide a pathway for the pathogen to transfer between cropping seasons due to the cross-infectivity of *P. brassicae* between oilseed rape and other *Brassica* species (Maddock et al., 1981; Evans et al., 2003). Therefore, it is necessary to put more emphasis on host resistance in LLS management practices and produce cultivars that have a greater economic return to sustain the production of oilseed rape, which is the third largest arable crop in the United Kingdom (DEFRA, 2020). The development of oilseed rape cultivars with good levels of field resistance against *P. brassicae* can provide economical means of disease control, especially for farmers with small to medium-sized arable farming areas.

Even though the average LLS resistance rating of oilseed rape cultivars has increased in recent years^1^, frequent recent epidemics of LLS indicate that the currently available cultivar resistance is inadequate to achieve successful control of this disease (CropMonitor, 2016). There has been little knowledge on the genetic basis of host resistance against *P. brassicae* (Karandeni Dewage et al., 2021) with only three published studies on mapping qualitative or quantitative resistance genes (Pilet et al., 1998; Bradburne et al., 1999; Boys et al., 2012). Moreover, sexual reproduction of *P. brassicae* could lead to the development of new virulent strains, rendering the resistance genes ineffective (Boys et al., 2007; Karandeni Dewage et al., 2018). Therefore, diversification of the resistance sources is essential to achieve effective and prolonged control of LLS through host resistance. Improvement of cultivar resistance through selective breeding requires sufficient genetic diversity to be present within the current gene pool. Nevertheless, continuous selection of plant material for specific traits can cause genetic bottlenecks, resulting in reduced genetic diversity in the primary gene pool (Doebley et al., 2006). Narrow gene pools can make crop species more vulnerable to emerging pests and pathogens and reduce the potential for improving crop productivity (Hyten et al., 2006). In such cases, genetic variations present in external gene pools provide plant breeders with an opportunity to improve crop cultivars by incorporating various traits for which there is insufficient diversity in the primary gene pools (Boyd et al., 2013).

Oilseed rape has been known for its narrow genetic diversity caused by strong selection for various traits in its breeding history (Snowdon and Iniguez Luy, 2012). For example, the process of developing modern double-low cultivars (low seed glucosinolate and low erucic acid contents) is considered to have caused genetic bottlenecks in current *B. napus* gene pools. However, being a natural hybrid between *B. rapa* and *B. oleracea* (Chalhoub et al., 2014), the compatibility between these two ancestral species (secondary gene pool) of *B. napus* enables introgression of new sources of resistance from these external gene pools into cultivated *B. napus*. Compared to *B. napus*, *B. rapa*, and *B. oleracea* are considered to have higher genetic diversity and they have proved to be effective in providing new resistance genes against other important pathogens of oilseed rape (Neik et al., 2017; Robin et al., 2017; Katche et al., 2019). Experimental work described in this article has focused on analysing a doubled haploid (DH) population of oilseed rape, derived from the ancestral genomes of *B. napus*, as a potential source of resistance against the LLS pathogen *P. brassicae* and understanding the operation of host resistance against this pathogen.

## Materials and Methods

### Plant Material

A progeny of DH lines (Q population consisting of a total of 92 lines) derived from an F1 cross between synthetic *B. napus* (*B. oleracea atlantica* X *B. rapa oleifera* “29”) and oilseed rape cv. Tapidor (European winter-type cultivar with a strict vernalisation requirement and low seed erucic acid and low glucosinolate content) (Mithen and Magrath, 1992; Smooker et al., 2011) was used in this study. Additionally, oilseed rape cultivars Canberra [UK Agriculture and Horticulture Development Board (AHDB) recommended list (RL) resistance rating 7 (2007/08), resistant], Cuillin [RL resistance rating 8 (2014/15), resistant], Marathon [RL resistance rating 5 (2016/17), susceptible], Bristol [RL resistance rating 2 (1996/97), susceptible], Imola (characteristic black flecking resistance phenotype against *P. brassicae* infection, Boys et al., 2012), and Tapidor and *B. rapa oleifera* “29” (A-genome parent of synthetic *B. napus*) were included in the phenotyping experiments. The AHDB RL ratings (resistance rating for LLS on 1–9 scale, where nine is most resistant), which are available for commercial cultivars, were taken from the most recent records available (given in parentheses next to the RL rating).

### Phenotyping of Resistance Against *Pyrenopeziza brassicae* in the Q Doubled Haploid Population

The Q DH population was assessed for its resistance against *P. brassicae* in three separate experiments to represent different *P. brassicae* populations (mixture of isolates collected from diseased leaves from oilseed rape crops) and different environmental conditions. These consisted of two glasshouse experiments (GH) and one controlled-environment (CE) experiment. Each experiment included appropriate resistant/susceptible control cultivars. The numbers of Q DH lines and resistant/susceptible control cultivars included in each of the three phenotyping experiments are given in Table 1. Different *P. brassicae* populations that originated from England or Scotland were used in the three experiments (Table 1). *Pyrenopeziza brassicae* conidial suspensions were prepared by selecting diseased oilseed rape leaves with clear LLS symptoms and incubating them at 4°C for 5 days in sealed polyethylene bags with a layer of dampened paper towel to increase humidity to induce sporulation. Leaves were then washed with sterile distilled water to produce conidial inoculum. Conidial suspensions were filtered through sterile Miracloth (Calbiochem, United States), and the concentration of each spore suspension was measured using a haemocytometer. Spore concentration was adjusted with sterile distilled water to 10^5^ spores/ml for the glasshouse experiments and 10^4^ spores/ml for the CE experiment and suspensions were stored at –20°C until needed.

**TABLE 1:** Summary of phenotyping experiments used to assess resistance against *Pyrenopeziza brassicae* in the *Brassica napus* Q doubled haploid (DH) population.

Experiment	Number of Q DH lines^a^	Control cultivars^b^	Origin of *Pyrenopeziza brassicae* inoculum^c^	Inoculum concentration (spores/ml)	Light leaf spot severity (% leaf area affected) (range)
Glasshouse 1 (GH1)	84	Marathon (S), Bristol (S), Cuillin (R), Imola (R), Tapidor, *Brassica rapa oleifera* “29”	Morley, Norfolk, England	1 × 10^5^	0–83%
Glasshouse 2 (GH2)	78	Bristol (S), Charger (S), Imola (R)	Aberdeen, Scotland	1 × 10^5^	0–63%
Controlled environment (CE)	89	Canberra (R)	Harpenden, Hertfordshire, England	1 × 10^4^	0–46%

*^a^Q DH population consists of 92 lines in total. From GH1 and GH2, 77 and 70 lines were taken into the final data analysis, respectively. Therefore, there were 70 lines in common between the three experiments.*

*^b^Each experiment included resistant (R)/susceptible (S) control cultivars. Additionally, GH1 experiment included two of the parental lines of the Q DH population; B. rapa oleifera “29” (A-genome parent of the synthetic B. napus) and cv. Tapidor.*

*^c^Plants were inoculated with P. brassicae populations (conidial suspensions collected from diseased leaves from oilseed rape crops in England or Scotland).*

Glasshouse experiments were arranged in an alpha design generated using an alpha design generator (Parsad et al., 2007) as it was not possible to assess all the lines/cvs in one experiment due to space limitations. Q DH lines were divided into small batches and assessed at different occasions within each glasshouse experiment (four and three batches in the first and second glasshouse experiment, respectively). Resistant/susceptible control cultivars and a Q DH line (Q12) were repeated on each occasion in GH1 and GH2 to monitor the uniformity of experimental conditions. Plants were grown in 9 cm diameter pots until they reached growth stage 1,4–1,5 (plants have five true leaves) (Sylvester-Bradley et al., 1984). Five replicate plants were included for each Q DH line and control cultivar. At growth stages 1,4–1,5, plants were spray-inoculated with *P. brassicae* conidial suspensions using a 50 ml travel spray bottle (Boots, United Kingdom) until all the leaves were evenly and fully covered with fine droplets of the spore suspension. After inoculation, plants were covered with a polyethylene cover for 48 h to maintain high humidity to facilitate spore germination and infection. Glasshouse conditions were maintained at 16°C/14°C day/night temperatures with natural daylight and supplemented by a 12 h photoperiod. At 24 days post-inoculation (dpi), plants were destructively harvested by cutting at the stem base above the compost surface, individually placed in polyethylene bags with a dampened paper towel, and incubated at 4°C for 5 days to induce sporulation. The disease assessment was made by visually estimating the percentage leaf area covered with *P. brassicae* acervuli (sporulation). The presence or absence of a necrotic response was also recorded for each plant.

In the CE experiment, plants were first grown in 7 cm diameter pots and maintained in a glasshouse at 20°C for 3 weeks. Four replicate plants were included for each Q DH line and the control cultivar. At growth stages 1,4–1,5, plants were spray-inoculated using an aerosol sprayer (Chrom Atomiser, Camlab; Cambridge, United Kingdom) until drops ran off the leaves. After inoculation, plants were individually covered with polyethylene bags (26 cm × 38 cm) to maintain high humidity to facilitate spore germination and infection and kept in a CE room at 16°C with a 12 h photoperiod. Bags were removed 48 h after inoculation and were replaced 14 days later for the final week before disease assessment. LLS severity on each plant was assessed by visually estimating the percentage leaf area covered with *P. brassicae* acervuli.

### Statistical Analysis and Mapping of Quantitative Trait Locus for Resistance Against *Pyrenopeziza brassicae*

Light leaf spot severity data (% leaf area covered with *P. brassicae* sporulation) were subjected to ANOVA using GENSTAT statistical software for Windows (Payne et al., 2011). Arcsine transformation of the percentage of leaf area with sporulation was made with an arcsine formula in Excel before ANOVA was done, so that the variance was more homogeneous across treatments and measurements were normally distributed. For each glasshouse experiment, data generated by the alpha design experiments were combined and the effect of experiment was analysed as a factor in the ANOVA using control cultivars and Q DH lines, which were included in each experiment (batch). For analysis of the relationships between LLS severities measured in each of the three phenotyping experiments, simple linear regression analyses were done using calculated means for different Q DH lines and cultivars.

The linkage map and marker data for the Q DH population have been described previously (Smooker et al., 2011). The genetic linkage map of the Q DH population comprised of 358 simple sequence repeats (SSR) markers over 19 linkage groups with a total genetic distance of 1,381 cm. QTL mapping was implemented with QTL cartographer version 2.5 (Wang et al., 2012) using quantitative disease severity (% leaf area with *P. brassicae* sporulation) data within each individual experiment and across all three experiments using combined data. Initial genome scans for marker-trait associations were done using single marker analysis to identify possible QTLs. The results obtained from single marker analysis were further refined using interval mapping (IM) (Haley and Knott, 1992). Genome-wide QTL threshold was determined by permutation analysis using 1,000 iterations corresponding to a significance level of α = 0.10. Support interval for each QTL was determined based on the decrease in 1.5-logarithm of the odds (LOD) on either side of the LOD maximum (Silva et al., 2012). Since IM may be affected by the skewed distribution of phenotype data, QTL positions and the effects were confirmed with transformed data. The binary (categorical) phenotype data for necrosis obtained from the two glasshouse experiments (presence or absence of a necrotic response) were analysed using the non-parametric Kruskal–Wallis test. QTLs detected by IM were visualised on the linkage map of the Q DH population using MapChart software (version 2.32) (Voorrips, 2002) with manual editing.

### Assessment of the Sub-Cuticular Growth Phase of *Pyrenopeziza brassicae* in Q Doubled Haploid Lines

Four Q DH lines (Q04, Q38, Q69, and Q83), based on the amount of *P. brassicae* sporulation and presence of a necrotic response observed in phenotyping experiments, and oilseed rape cultivars Bristol and Imola were selected. Plants were grown in 9 cm diameter pots under controlled-environment conditions (FITOCLIMA D1200, ARALAB, Rio de Mouro, Portugal) with a 12 h photoperiod, 60% relative humidity, and 20°C/18°C day/night temperatures, respectively. Plants were arranged in a randomised complete block design generated using Experimental Design Generator and Randomiser (EDGAR) (Brown, 2004). At growth stage 1,4–1,5 (five leaves unfolded) (Sylvester-Bradley et al., 1984), plants were point-inoculated at four “marked” points on adaxial surfaces of each of the fourth and fifth true leaves using sterilised Whatman no. 1 filter papers (cut into *c.*0.8 mm × 0.8 mm squares) immersed in a *P. brassicae* conidial suspension (10^5^ spores/ml). After inoculation, plants were covered with a polyethylene cover for 48 h to maintain high humidity. Inoculated plants were maintained in the controlled environment cabinet with a 12 h photoperiod, 60% relative humidity, and 16°C/14°C day/night temperatures, respectively.

Plants were sampled at 0, 3, 7, 14, and 24 dpi, and the fourth and fifth true leaves were used for the analysis of sub-cuticular growth of *P. brassicae* using quantitative PCR (qPCR) with species-specific primers (Karolewski et al., 2006) and scanning electron microscopy (SEM), respectively. For qPCR analysis, the fourth true leaf to appear was removed from each plant and 2 cm diameter leaf discs were cut from each inoculation point. Leaf discs were individually placed in 2 ml tubes, frozen at –20°C, and freeze-dried. Samples were processed in a Fastprep machine (MP Biomedicals, United Kingdom) with three metal beads (3 mm diameter) until leaf discs were ground to a fine powder. DNA extraction and quantification of *P. brassicae* DNA was done according to the method described in Boys et al. (2012) with minor modifications (DNA samples were diluted to a final concentration of 20 ng/μl and five standards, each containing known quantities of *P. brassicae* DNA ranging from 1 pg to 10 ng, were used in qPCR). Quantitative PCR data were analysed by simple linear regression of *P. brassicae* DNA content against the days post-inoculation (dpi). Since the amount of *P. brassicae* DNA in leaf tissues showed an exponential increase with time after inoculation, data were transformed by taking the common logarithm (log_10_) of the original measurements. A position and parallelism regression analysis was used to analyse the differences in the increase in the amount of *P. brassicae* DNA over time between the six lines/cultivars included in this experiment. All the analyses were done using GENSTAT statistical software for Windows (Payne et al., 2011).

Scanning electron microscope (SEM) analysis of leaf samples of the Q DH lines collected at different time points after inoculation was done using the Bioimaging facility at Rothamsted Research, Harpenden, United Kingdom.^2^ Pieces of leaves (*c.* 4 mm × 4 mm) were obtained from the inoculation points using a sterile blade and were prepared according to standard operating procedures of the SEM instrument (JEOL JSM-6360, JEOL Ltd., United Kingdom) at Rothamsted Research for examination and recording images.

## Results

### Phenotyping of Resistance Against *Pyrenopeziza brassicae* in the Q Doubled Haploid Population

**FIGURE 1:**
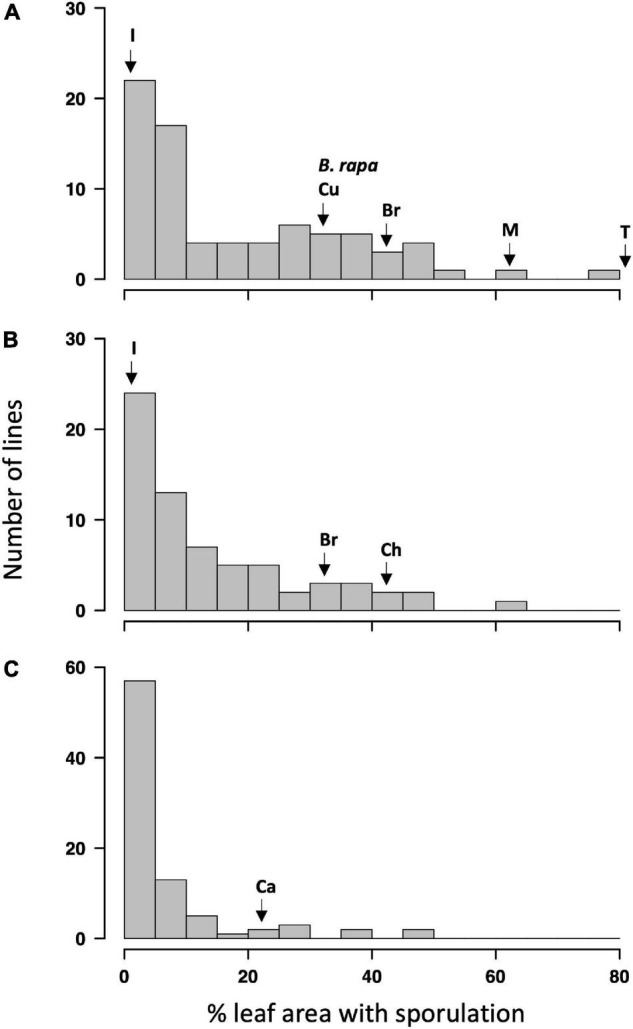
Frequency distribution of light leaf spot severity (% leaf area affected) in the Q doubled haploid (DH) population in three phenotyping experiments. **(A)** Glasshouse experiment 1 (GH1), **(B)** glasshouse experiment 2 (GH2), **(C)** controlled-environment experiment (CE). Arrows indicate the position of parental lines [*B. rapa olifera* “29” (*B. rapa*) and Tapidor (T)] and the resistant [Imola (I), Cuillin (Cu), and Canberra (Ca)] or susceptible [Bristol (Br), Charger (Ch), and Marathon (M)] control *Brassica napus* cultivars in the phenotypic distribution.

The ANOVA indicated a significant effect of genotypes (*p* < 0.01) on the LLS severity. The effect of the batch experiment was not significant within GH1 and GH2 (*p* > 0.69, Supplementary Table 1). Distribution of resistance against *P. brassicae*, measured using % leaf area covered with pathogen sporulation, among Q DH lines is illustrated in Figure 1. The phenotype distribution showed positive skew toward resistance in all three experiments. Significant positive correlations were observed between the experiments (Supplementary Table 2; *p* < 0.01), indicating consistency in disease scoring methodology across experiments. The correlation was particularly good between the two glasshouse experiments, and the overall LLS severity in these two experiments appeared to be greater [ranging from 0 to 83% and from 0 to 63% leaf area affected in the first (GH1) and second (GH2) glasshouse experiments, respectively] compared to that of the CE experiment (ranging from 0 to 46%). Some of the Q DH lines had disease severities smaller than or equal to those of the resistant control cultivars (cvs Imola and Cuillin) in GH1 and GH2 experiments, and most of the Q DH lines had disease severities smaller/equal to those of the resistant control (cv. Canberra) in the CE experiment. Thirty-nine lines in GH1 and 27 lines in GH2 showed no significant difference from the disease severity observed on cv. Imola (0.9 and 0% leaf area with sporulation in GH1 and GH2, respectively) (*p* < 0.05). The two parental lines included in the GH1 experiment differed significantly in LLS severity (*p* < 0.05). Of these, the A-genome parent of the synthetic *B. napus*, *B. rapa oleifera* “29” showed an average of 32.5% leaf area with sporulation, which is similar to that of cv. Cuillin. In contrast, cv. Tapidor showed extreme susceptibility to *P. brassicae* with an average of 83% leaf area with sporulation.

**FIGURE 2:**
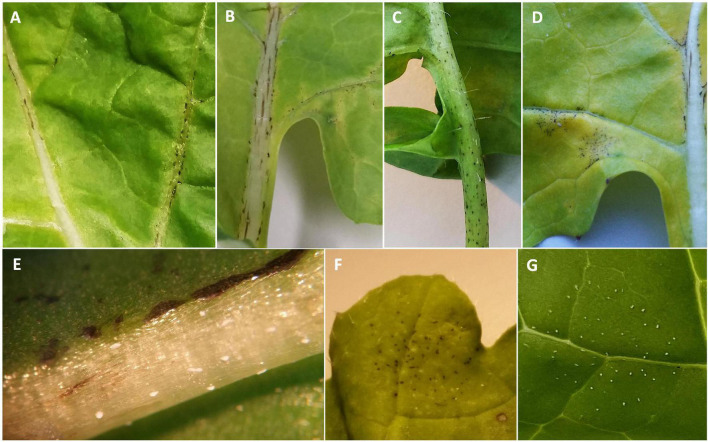
Necrotic responses observed in different *B. napus* lines from the Q DH population. **(A)** Q88, **(B)** Q60, **(C)** Q83, **(D)** cv. Imola, **(E)** Q64 with necrosis and occasional acervuli on the midrib, **(F)** Q33 with necrosis on leaf lamina in concentric rings, **(G)** susceptible line Q38 with concentric ring-like sporulation patterns characteristic of *Pyrenopeziza brassicae*.

**FIGURE 3:**
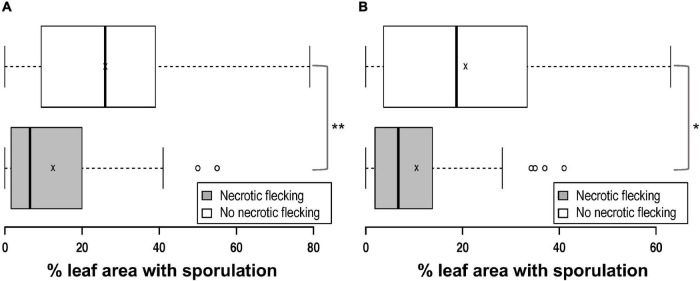
Box plots comparing light leaf spot severity of Q DH lines with or without the presence of black necrotic flecking observed in two glasshouse experiments. **(A)** GH1, **(B)** GH2. Q DH lines inoculated with *P. brassicae* appeared to have a variation in the light leaf spot severity (% leaf area with sporulation) with or without the presence of necrosis. In each plot, the centre lines crossing the boxes denote median values, box edges represent the lower 25% and the upper 75% quartiles, x denotes the mean value and error bars represent minimum and maximum values. There was a significant difference in the median light leaf spot severity values between the two groups (*p < 0.05; **p < 0.01).

In addition to the varying numbers of *P. brassicae* acervuli that appeared with or without lesion formation, some of the Q DH lines showed a necrotic response against *P. brassicae* that started to appear *c.* 10–14 dpi (Figures 2A–C). These responses were mainly observed on the leaf veins, midribs, and along petioles, and elsewhere on the leaf lamina (leaf blade). This was similar to the necrosis observed on cv. Imola (Figure 2D) that is known to contain a major-gene locus for resistance against *P. brassicae*. However, cv. Imola was consistent in producing zero to very little (>1% area affected) sporulation (mainly confined to leaf veins, the midrib, and petioles) in different experiments, whereas the Q DH lines with necrosis showed a great variation in sporulation. For example, some of the Q DH lines appeared to have large numbers of acervuli in the presence of a less intense necrotic response. Some lines showed limited sporulation confined only to the leaf veins and the midribs (Figure 2E). Necrotic flecking on the leaf lamina appeared in concentric rings (Figure 2F) which resembled concentric ring-like sporulation patterns characteristic of susceptible interactions (Figure 2G). In the GH1 experiment, 41 out of 77 lines produced a necrotic response, whereas in GH2 experiment, 46 out of 70 lines produced necrosis. Of these, six lines that had necrosis in GH1 did not produce necrosis in GH2, and nine lines that did not have necrosis in GH1 produced necrosis in GH2. Comparisons of the LLS severities between Q DH lines with or without a necrotic response using qualitative assessments (presence or absence) made in GH1 and GH2 experiments are illustrated in Figure 3. According to Shapiro-Wilk W statistics, LLS severity distributions showed deviation from normality (*p* < 0.05) in both groups. The difference in the median values of % leaf area covered with acervuli between Q DH lines with or without a necrotic response was statistically significant in GH1 (*p* < 0.01) and GH2 (*p* < 0.05) experiments.

### Mapping of Quantitative Trait Locus for Resistance Against *Pyrenopeziza brassicae*

**FIGURE 4:**
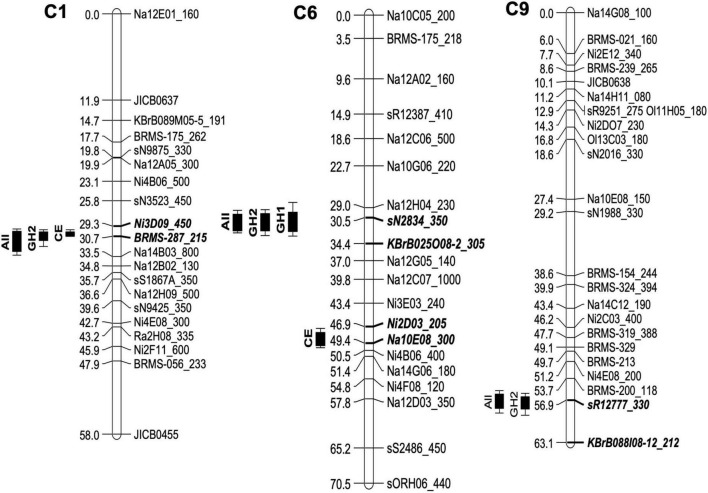
Quantitative trait loci (QTLs) for resistance against *P. brassicae* in the *B. napus* Q DH population detected using three phenotyping experiments. Phenotyping of the Q DH population was done using two glasshouse experiments (GH1 and GH2) and a CE experiment with each involving different *P. brassicae* populations. Four QTLs were identified across three linkage groups, C1, C6, and C9 using QTL analysis within individual experiments. Combined data across all three experiments identified three QTLs (labelled “All”) co-locating with those identified within individual experiments. On the left, QTL positions are marked with LOD support intervals and flanking markers for each QTL are indicated by bold, italicised letters on the linkage maps.

Single marker analysis, which was used as the initial approach for the genetic mapping of the resistance against *P. brassicae*, indicated several loci with significant marker-trait associations. IM analysis within individual experiments identified four QTLs for resistance against *P. brassicae* across three linkage groups in the genetic linkage map for the Q DH population. Summary of the QTLs detected in each experiment, including maximum values of LOD scores, QTL positions, the percentage of phenotypic variance explained, and the estimate of QTL effects, is given in Table 2. Identification of QTL positions and effects were compared between transformed and untransformed data. This indicated similar results except for one QTL maximum on linkage group A10 for which the LOD score was less than the significant threshold with untransformed data. This QTL was removed from further analysis. One major QTL exceeding the LOD threshold was detected in the GH1 experiment located on the linkage group C6 (maximum LOD at 31.5 cM), accounting for 33.8% of the phenotypic variance. In comparison, three and two QTLs were detected in GH2 and CE experiments, respectively. The QTLs identified in the GH2 experiment were located on the linkage groups C1 (maximum LOD at 30.3 cM), C6 (maximum LOD at 31.5 cM), and C9 (maximum LOD at 57.0 cM), accounting for between 24.31 and 52.74% phenotypic variance. The two QTLs detected in the CE experiment included a major QTL located on the linkage group C1 (maximum LOD at 30.3 cM), accounting for 69.37% of the variance identified, and a QTL with a relatively small effect on the linkage group C6 (maximum LOD at 49.0 cM), accounting for 20.23% of the variance identified. Comparing the QTLs identified within different experiments (Figure 4), one of the QTLs on linkage group C6 was detected in both GH1 and GH2 experiments, and the QTL on linkage group C1 was detected in both GH2 and the CE experiments. For the GH1 experiment, a putative QTL co-located with the QTL on linkage group C1 identified in GH2 and CE experiments was detected with a LOD score of 3.1 that was just below the significance threshold (LOD = 3.3). QTL analysis for the combined data across all three experiments detected three QTLs co-located with those identified within individual experiments. These included QTLs located on the linkage groups C1 (maximum LOD at 30.4 cM), C6 (maximum LOD at 31.4 cM), and C9 (maximum LOD at 56.8 cm) (Figure 4), accounting for 37.31, 37.22, and 22.83% of phenotypic variance, respectively.

**TABLE 2:** Quantitative trait loci (QTLs) detected across three phenotyping experiments for resistance against *P. brassicae* in the *B. napus* Q DH population.

Experiment^a^	Linkage group^b^	QTL position (cM)^c^	Support interval (cM)^d^	Peak LOD	*R*^2^ (%)^e^	Additive effect	QTL significance^f^
GH1	C6	31.5	29.3–32.9	3.4	33.8	10.98	***
GH2	C1	30.3	30.3–31.8	5.5	52.74	12.03	****
	C6	31.5	29.6–32.9	3.5	34.59	9.15	***
	C9	57.0	56.2–58.6	4.1	24.31	7.54	****
CE	C1	30.3	30.1–30.5	7.4	69.37	12.21	**
	C6	49.0	47.6–49.9	3.3	20.23	–4.77	***

*^a^DH population was phenotyped in two glasshouse experiments (GH1 and GH2) and a controlled-environment experiment (CE), and QTL was determined within each individual experiment. A summary of the phenotyping experiments is given in Table 1.*

*^b^Linkage groups are labelled according to the standard chromosome/linkage group nomenclature of B. napus (A1–A10 and C1–C9) agreed by the Multinational Brassica Genome Project (MBGP) Steering Committee (http://www.brassica.info/resource/maps/lg-assignments.php).*

*^c^Flanking markers for each QTL have been indicated in Figure 4.*

*^d^LOD-1.5 support interval, which has a confidence interval close to 95%, is given for each of the QTL detected.*

*^e^% phenotypic variance explained by the QTL.*

^f^Significance based on individual marker-trait association. Significance at the 1, 0.1, and 0.01% levels are indicated by **, *** and ****, respectively.

Second QTL analysis was done for GH1 and GH2 experiments by taking sub-populations of Q DH lines based on the presence or absence of necrosis with the intention of distinguishing QTL effects related to these two phenotype groups. According to the results, only the QTL identified on linkage group C1 remained significant in GH2 for the sub-population of Q DH lines without necrosis. The QTL on the linkage group C9 was also retained, with a small decrease in the LOD score. The same phenomenon was observed in GH1, where the putative QTL maximum on the linkage group C1 was detected with the sub-population of Q DH lines without necrosis. Therefore, it is possible that the QTLs on the linkage groups C1 and C9 contribute to the reduced sporulation without necrosis. QTL mapping of the sub-population of DH lines with necrosis retained the QTL maximum on linkage group C6 with slightly reduced LOD scores for both GH1 and GH2, while showing a considerable loss of QTL maxima on the linkage group C1. The overall phenotypic variance in the sub-population of DH lines with necrosis may be attributed to the combined effects of QTLs (i.e., those related to reduced sporulation and necrosis). Considering the QTL maxima identified in the sub-population, it can be suggested that the QTL on the linkage group C6 contributes more toward the phenotypic variance in the group of DH lines with necrosis. However, no significant interactions with marker data exceeding the QTL threshold were detected for the binary data on necrosis.

### Assessment of the Sub-Cuticular Growth Phase of *Pyrenopeziza brassicae* in Q Doubled Haploid Lines

**FIGURE 5:**
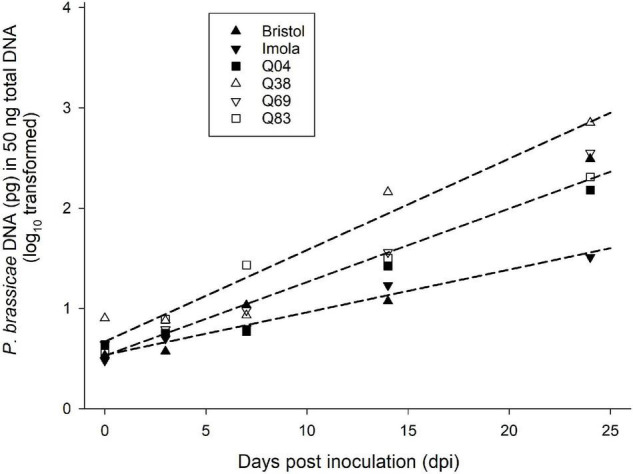
Change with time in amount of *P. brassicae* DNA in selected *B. napus* Q DH lines and control cultivars. In a controlled-environment experiment, four Q DH lines differing in resistance and oilseed rape cultivars Bristol and Imola were point-inoculated with a *P. brassicae* conidial suspension. Amounts of *P. brassicae* DNA in leaf samples taken from the points of inoculation between 0 and 24 dpi were quantified using qPCR. Position and parallelism regression analysis was used to analyse the difference in the increase of DNA with time between the six lines/cultivars. Data were best fitted by three regression lines and there were three distinct groups based on the difference in the increase of DNA with time: group 1 contained Q38 (*y* = 0.09*x* + 0.67), group 2 consisted of Q04, Q83, Q69, and cv. Bristol (*y* = 0.07*x* + 0.53) and group 3 contained cv. Imola (*y* = 0.04*x* + 0.53).

Using the results obtained in the three phenotyping experiments (GH1, GH2, and CE), four Q DH lines were selected to represent differences in the amounts of sporulation and the necrosis observed. These included Q83 with an average of <1% leaf area affected with sporulation in the presence of necrosis, Q4 with <5% leaf area with sporulation in the presence of necrosis, Q69 with <2% leaf area with sporulation without necrosis, and Q38 with >59% of leaf area with sporulation without necrosis. Imola and Bristol were the resistant and susceptible control cultivars, respectively. Analysis of *P. brassicae* DNA content in leaf discs taken from the points of inoculation showed a significant increase between 0 and 24 dpi in all the lines and cultivars. There were significant differences between lines/cultivars (*p* < 0.05). Position and parallelism regression analysis indicated three distinct groups based on the difference in the increase in *P. brassicae* DNA over time: group 1 contained Q38, group 2 consisted of Q04, Q83, Q69, and cv. Bristol, and group 3 contained cv. Imola (Figure 5). Line Q38, which developed large numbers of *P. brassicae* acervuli in all three phenotyping experiments (GH1, GH2, and CE), had the greatest amount of *P. brassicae* DNA and the greatest rate of increase over time. Cultivar Imola had significantly less *P. brassicae* DNA than the rest of the lines/cultivars and the amount of DNA increased over time at a slower rate.

**FIGURE 6:**
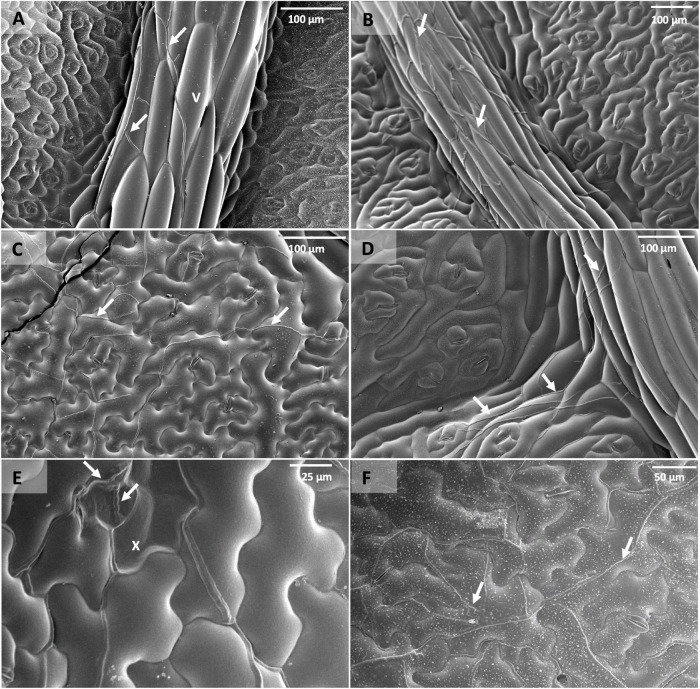
Scanning electron micrographs of leaf discs taken from *B. napus* Q DH lines point-inoculated with *P. brassicae* conidia in a controlled environment experiment. **(A)** Q83 at 7 dpi, **(B)** Q4 at 7 dpi, **(C)** Q38 at 7 dpi, **(D)** Q04 at 7 dpi showing *P. brassicae* hyphae following leaf vein (v) branching patterns, **(E)** epidermal cell collapse (x) on Q83 associated with *P. brassicae* hyphae, **(F)** Q4 at 24 dpi. Arrows indicate *P. brassicae* hyphae growing in sub-cuticular spaces of leaf vein and leaf lamina tissues.

Scanning electron micrographs also indicated that *P. brassicae* was capable of infecting and colonising all the lines selected in this experiment even though the extent of sub-cuticular hyphal growth varied among them (Figure 6). Lines Q83, Q4, and Q69 had hyphae growing more prominently along the leaf veins at early time points (i.e., 3–7 dpi). In addition, sub-cuticular hyphal growth on these lines appeared to follow the branching patterns of the main and lateral veins. This observation was consistent with the patterns of necrosis and acervuli production on the resistant lines. As the pathogen colonisation progressed with time (i.e., 14 dpi), the hyphae appeared to spread out onto the leaf lamina to a certain extent and more fungal biomass could be observed on the leaf lamina at *c.* 24 dpi. In addition, epidermal cell collapse associated with hyphae was observed on the Q DH lines with necrosis. In contrast, extensive hyphal growth could be observed on both the leaf veins and leaf lamina of the susceptible line Q38 from 7 dpi with more hyphae branching out from leaf veins onto the leaf lamina.

## Discussion

This article reports identification of new QTLs for resistance against *P. brassicae* derived from a *B. napus* secondary gene pool (ancestral genomes). Results from the phenotypic and genetic analysis of host resistance against *P. brassicae* provided good evidence for the segregation of resistance against *P. brassicae* in the Q DH population. Q DH lines differed from each other in their ability to limit *P. brassicae* asexual sporulation. There were significant differences in the % leaf area covered with sporulation between different lines. Reduced *P. brassicae* sporulation appeared to be present with or without a necrotic response and there was a significant difference between the two groups in the median values of % leaf area covered with acervuli. The concentric ring-like arrangement of the necrotic spots on the leaf lamina may indicate that *P. brassicae* asexual sporulation, which occurs in concentric rings in susceptible responses (Karandeni Dewage et al., 2018), is prevented by operation of the host resistance in these lines. This suggests that host recognition occurs at a late stage (*c.* 10–14 dpi) of *P. brassicae* colonisation, possibly during the asexual sporulation phase. However, the variation in amount of sporulation, observed between Q DH lines that gave a necrotic response upon pathogen recognition, suggests that there may be a background resistance or other resistance genes segregating in this population that affect the overall level of resistance to the pathogen.

Of the parental lines, neither the synthetic *B. napus* nor the C sub-genome parent of the synthetic *B. napus* (*Brassica oleracea altantica*) were available to test in our experiments. However, in the GH1 experiment, we were able to include *B. rapa olifera* “29” (A sub-genome parent of the synthetic *B. napus*) and cv. Tapidor, which showed moderate to high percentage leaf area with *P. brassicae* asexual sporulation without a necrotic response. According to these observations, it can be speculated that most of the favourable alleles for resistant QTLs were likely to have been contributed by C sub-genome parent *via* synthetic *B. napus*. The Q DH population is known to have an asymmetric distribution of marker polymorphisms between the A and C sub-genomes with allelic diversity diverted more toward the C sub-genome (Smooker et al., 2011). This seems to be true for the segregation of resistance against *P. brassicae* in the Q DH population, considering the distribution of QTLs on the C sub-genome.

In three phenotyping experiments, four QTLs were identified with moderate to large QTL effects. Linkage groups C1 and C6 appeared to have co-locating QTL stable across GH2/CE and GH1/GH2 experiments, respectively. Of these, the QTL on the linkage group C1 appeared to have a major effect on limiting *P. brassicae* asexual sporulation. QTL analysis is considered as the initial step toward marker-assisted selection (MAS) in plant breeding, and for a particular QTL to be effective in a plant breeding programme, it is important to confirm the repeatability and the efficiency of the QTLs in different environments (Collard et al., 2005). Accurate identification of QTL depends on the quality of the phenotyping data and the robustness of the linkage map. A significant positive correlation of the phenotype data between the different experiments indicated consistency in disease scoring methodology across experiments. However, it should be noted that the relatively small population size used in this study probably resulted in false negatives, particularly in detecting QTLs with relatively small effects, along with possible over-estimation of the QTL effects.

There seem to be several QTLs contributing to the overall phenotypic variation. QTL-mediated resistance associated with reduced *P. brassicae* sporulation or leaf necrosis is probably controlled by different *B. napus* resistance loci. However, no significant QTL was detected when the binary data for necrosis were used as a phenotype, which could have been due to the genetic complexity of this phenotype. All the experiments were done with *P. brassicae* populations (mixtures of isolates) and some of the lines that had necrosis in GH1 did not produce necrosis in GH2 and *vice versa*. It is possible that there were different pathogen races or effectors recognised by the host. Furthermore, there seemed to be differences in the intensity of black flecking observed in different lines, indicating that the expression of this necrosis phenotype may be affected by other QTLs for resistance, or that this could be a component of a network of host responses. When we separate the DH lines based on the presence or absence of necrosis, the lines with necrosis contain phenotypic variation attributed to the combined effect of different loci, whereas the effect of necrosis is eliminated from the lines without necrosis. This may provide a possible genetic explanation for the significantly smaller median LLS severity values observed in the group of DH lines that showed necrosis compared to the group without necrosis.

Similar phenotype classes for resistance against *P. brassicae* have been explained by Bradburne et al. (1999) in a DH population of *B. napus*. Bradburne et al. (1999) reported two major genes for resistance against *P. brassicae*. Linkage analysis positioned the gene corresponding to “no asexual sporulation” (*PBR1*) on linkage group A1 and the gene responsible for “dark necrotic flecking” (*PBR2*) on linkage group C6. The QTL on linkage group C6 has been localised toward the centre of the linkage group in both Bradburne et al. (1999) and the present study. There is a possibility that both the studies refer to the same resistance locus. However, the linkage map published by Bradburne et al. (1999) contains limited information with only two restriction fragment length polymorphism (RFLP) markers on C6. There are no common markers between the two studies, which are necessary to create a consensus map between different populations to enable the comparison of QTLs across different studies. Therefore, it is difficult to draw a conclusion with the information currently available. Regarding the work reported by Pilet et al. (1998) on quantitative resistance against *P. brassicae*, QTLs were detected mostly on the A sub-genome (linkage groups A2, A6, A7, and A9) and one QTL was detected on the C sub-genome (linkage group C4), whereas the present study identified QTLs on linkage groups C1, C6, and C9.

Using a DH population derived from the material described by Bradburne et al. (1999) and Boys et al. (2012) reported a single locus for resistance corresponding to the black flecking phenotype (*PBR2*) that mapped to the bottom end of chrA1. The second major gene (*PBR1*, corresponding to the absence of asexual sporulation) reported by Bradburne et al. (1999) was not identified and it has been suggested that *PBR1* might have been lost during the breeding process. In the present study, we have identified DH lines with little to no sporulation without necrosis. These lines can be used to further dissect the genetic basis of this resistant phenotype by developing a larger mapping population for fine-scale mapping. Availability of *Brassica napus* genomic resources offers new possibilities for the identification of host resistance genes and provides molecular tools to assist in marker-assisted selection (MAS) for disease resistance. There are a few *B. napus* genome sequences published, including the genome sequence of cv. Tapidor (Chalhoub et al., 2014; Bayer et al., 2017; Sun et al., 2017), which is one of the parental lines of the Q DH population. Sequencing of the flanking markers can be used to identify the corresponding genomic regions of the QTL on the *B. napus* genome, facilitating the identification of candidate resistance genes.

The Q DH population segregates for vernalisation and winter hardiness (Smooker et al., 2011), making it difficult to assess some of the lines directly in winter oilseed rape field experiments in the United Kingdom. Therefore, we chose to phenotype the Q DH population under controlled-environment and glasshouse conditions to enable the identification of different components of resistance without being affected by other characters segregating in this population. Instead of using single-spore isolates, plants were inoculated with a different *P. brassicae* population in each experiment, representing natural inoculum. In a separate study that assessed resistance against *P. brassicae* in different *B. napus* genotypes, selected Q DH lines have shown more resistance compared to that of commercial oilseed rape cultivars. In addition, the resistance in those lines appeared to be less sensitive toward the increasing virulence of *P. brassicae* isolates (Karandeni Dewage et al., 2021). This agrees with the stability of the resistance in Q DH lines across different *P. brassicae* populations observed in the present study. Host resistance QTLs that are stable across different *P. brassicae* populations are of particular importance to oilseed rape breeders.

Our results suggest that the resistance segregating in this DH population limits colonisation/sporulation by the pathogen rather than eliminating the pathogen. *Pyrenopeziza brassicae* was able to infect and colonise both resistant and susceptible Q DH lines and control cultivars with a significantly smaller rate of increase of pathogen biomass in resistant lines than that in susceptible lines. According to the qPCR data, three resistant Q DH lines were in the same group as cv. Bristol that had a significantly greater number of *P. brassicae* acervuli in phenotyping experiments. This also supports the suggestion that there may be resistance operating during the time of *P. brassicae* asexual sporulation. According to SEM, hyphal growth was more prominent along the leaf veins of resistant lines, especially at early time points. According to Boys et al. (2012), the amount of *P. brassicae* DNA was significantly greater in leaf vein/midrib tissues than elsewhere in the leaves of cv. Imola when samples were taken from the points of inoculation. This suggests that the pathogen was able to colonise more of leaf vein/midrib tissues than tissues of interveinal regions. It can be assumed that abundant vascular bundles in leaf veins provide the pathogen with more access to resources and hence support the extracellular colonisation of leaf veins, midribs, and petioles. This may also explain the presence of the black flecking phenotype, mainly along the leaf veins, midrib, or petioles, and the production of occasional acervuli along with these tissues in the case of resistant cultivars. There have been similar reports of other endophytic fungi with more affinity toward leaf vein/petiole colonisation (Photita et al., 2001; Toofanee and Dulymamode, 2002).

Even though LLS is considered as a major disease problem of oilseed rape in the UK with many epidemics since 1970s, little is known about this pathosystem in contrast to that of other important diseases, such as phoma stem canker (Boys et al., 2007; Karandeni Dewage et al., 2018). So far, there have been only two published studies on genetic mapping of major-gene-mediated resistance (Bradburne et al., 1999; Boys et al., 2012) and one study reporting quantitative resistance against *P. brassicae* in *B. napus* (Pilet et al., 1998). Therefore, new studies on qualitative and quantitative resistance against *P. brassicae* are invaluable to combat LLS to sustain oilseed rape production in the United Kingdom.

Quantitative trait locus mapping can be used to determine QTL effects, interactions between resistance genes, race-specificity of resistance, etc., providing insights into resistance against pathogens that has complex inheritance (Young, 1996; St. Clair, 2010). Even though QDR has been studied in several pathosystems, underlying molecular mechanisms of QDR are not well understood, with few studies reporting functional characterisation of resistance QTL. In this study, we were able to do detailed phenotyping of the resistance against *P. brassicae* originating from the ancestral brassica species and allocate the observed variation into its genetic components. Resistant lines identified can serve as pre-breeding material and the QTLs that are stable across different experiments could be utilised in MAS in oilseed rape breeding programmes to improve cultivar resistance against *P. brassicae* with further resolution of resistance QTLs. Associated markers can be used as a starting point for functional identification of the genes controlling resistance against *P. brassicae* that can contribute to our knowledge on mechanisms of partial resistance/QDR of crops against pathogens.

## Data Availability Statement

The raw data supporting the conclusions of this article will be made available by the authors, without undue reservation.

## Author Contributions

CKD, HS, Y-JH, and BF conceived the project. CKD and KC designed and did the experiments. AQ and CKD did the data analysis. RW contributed to the resources and assisted with analysis. CKD conceptualised and drafted the manuscript with additions and edits from BF, AQ, Y-JH, and HS. All authors read and approved the final manuscript.

## Conflict of Interest

The authors declare that the research was conducted in the absence of any commercial or financial relationships that could be construed as a potential conflict of interest.

## Publisher’s Note

All claims expressed in this article are solely those of the authors and do not necessarily represent those of their affiliated organizations, or those of the publisher, the editors and the reviewers. Any product that may be evaluated in this article, or claim that may be made by its manufacturer, is not guaranteed or endorsed by the publisher.

## Footnotes

1
https://ahdb.org.uk/rlarchive

2
https://www.rothamsted.ac.uk/bioimaging
