# Supplementary material for: Quantitative Trait Locus Mapping for Resistance Against Pyrenopeziza brassicae Derived From a Brassica napus Secondary Gene Pool
Source: Front Plant Sci. 2022 Feb 4;13:786189. doi: 10.3389/fpls.2022.786189 (PMC8854361; doi:10.3389/fpls.2022.786189)
Supplement: Supplementary file 1 [file Table_1.DOCX]

**Table S1:** Results of analysis of variance showing the effects of batch number (i.e. the effects of different environments in CE, GH1 and GH2) on the light leaf spot severity as percent leaf sporulation (arcsine transformed) including 70 Q DH lines replicated five times in each experiment.

| Factor | Degrees of freedom | Sum squares | Mean square | F value | Probability significance |
| --- | --- | --- | --- | --- | --- |
| Batch Numbers | 2 | 0.04331 | 0.02165 | 0.7 | 0.49 |
| Replication | 4 | 0.14723 | 0.03681 | 1.19 | 0.32 |
| Blocks | 45 | 4.57367 | 0.10164 | 3.29 | <0.01 |
| Q DH line | 69 | 19.05003 | 0.27609 | 8.93 | <0.01 |
| Residual | 260 | 8.0403 | 0.03092 |  |  |
| Total | 380 | 31.85453 | 0.08383 |  |  |
