# Supplementary material for: Quantitative Trait Locus Mapping for Resistance Against Pyrenopeziza brassicae Derived From a Brassica napus Secondary Gene Pool
Source: Front Plant Sci. 2022 Feb 4;13:786189. doi: 10.3389/fpls.2022.786189 (PMC8854361; doi:10.3389/fpls.2022.786189)
Supplement: Supplementary file 2 [file Table_2.DOCX]

**Table S2:** Correlation matrix for relationship between the light leaf spot severity assessments in different phenotyping experiments with the *B. napus* Q DH population.

| **Experiment** | **Correlation coefficient^a^** | | |
| --- | --- | --- | --- |
|  | CE | GH1 | GH2 |
| CE | 1 |  |  |
| GH1 | 0.34 | 1 |  |
| GH2 | 0.38 | 0.77 | 1 |

^a^ all correlations are significant at *P*<0.01
